# On the Opium of Lettuce

**Published:** 1801-11

**Authors:** 


### *On the Opium of Lettuce.*

DR. REDMAN COXE, of Philadelphia, has made a number of interefting experiments on the comparative virtues of the Opium obtained from common Lettuce and that from the Poppy. Some of thefe we propofe to lay before our Readers in a future Number; at prefent, we fhall extract the following important inference.

"All the fpecies of Lettuce contain the juice in a larger or fmaller proportion. The *lactuca sylvestris*, or *virosa* of Linnæus, contains it moft abundantly. That from which I obtained my opium was the *lactuca sativa*; it abounds in juice, and will ferve the double purpofe of cultivating for the table as well as for the fhop.

"I cannot avoid contrafting the fuperior advantages of the opium extracted from the Lettuce, above that procured from the poppy.

"Some judgment may be formed of the labour and expence attendant upon the cultivation of one acre of the poppy, by the account given by Mr. Kerr. He fays, 'An acre yields in the Eaft Indies, 60lb. of opium, which, at nine fhillings fterling (two dollars) per pound, is 27 *l.* an acre.' Now, at a moderate computation, it may be prefumed that *one half* of this fum is employed in the neceffary expences of ploughing, manuring, fowing, watering, collecting, &c. Say then, that 13*l.* 10*s.* are clear gain, (which muft be allowed to be a large proportion.) Now the *poppy* cannot be employed as an article of diet; whereas the *lettuce*, which grows here in the moft luxuriant manner, will amply repay the labour and expence (which at moft is trifling) attending its cultivation, by the fale of the fupernumerary plants taken up at an early period for diet, long before the developement of the opium principle. Here then the very labour employed has the double advantage of thinning the plants, thereby rendering the remainder more perfect; whilft it collects for the market fuch as have arrived to fufficient maturity for the table.

"The fale of thefe fupernumerary plants would, I conceive, at leaft, repay the labour, &c. attending their cultivation; and if the reft yielded, per acre, only 60lb. of opium, double the profit would arife from its cultivation above that of the poppy. The great abundance of the juice, however, and the luxuriance of the plant, render it highly probable that double that quantity, if not more, might be procured from the acre of ground."

Some

